# The Synergistic Effect of Triterpenoids and Flavonoids—New Approaches for Treating Bacterial Infections?

**DOI:** 10.3390/molecules27030847

**Published:** 2022-01-27

**Authors:** Natalia Wrońska, Michał Szlaur, Katarzyna Zawadzka, Katarzyna Lisowska

**Affiliations:** Department of Industrial Microbiology and Biotechnology, Faculty of Biology and Environmental Protection, University of Lodz, 12/16 Banacha Street, 90-236 Lodz, Poland; n37@o2.pl (M.S.); katarzyna.zawadzka@biol.uni.lodz.pl (K.Z.); katarzyna.lisowska@biol.uni.lodz.pl (K.L.)

**Keywords:** phytocompounds, ursolic acid, oleanolic acid, dihydromyricetin, antibacterial activity, cytotoxicity

## Abstract

Currently, the pharmaceutical industry is well-developed, and a large number of chemotherapeutics are being produced. These include antibacterial substances, which can be used in treating humans and animals suffering from bacterial infections, and as animal growth promoters in the agricultural industry. As a result of the excessive use of antibiotics and emerging resistance amongst bacteria, new antimicrobial drugs are needed. Due to the increasing trend of using natural, ecological, and safe products, there is a special need for novel phytocompounds. The compounds analysed in the present study include two triterpenoids ursolic acid (UA) and oleanolic acid (OA) and the flavonoid dihydromyricetin (DHM). All the compounds displayed antimicrobial activity against Gram-positive (*Staphylococcus aureus* ATCC 6538, *Staphylococcus epidermidis* ATCC 12228, and *Listeria monocytogenes* ATCC 19115) and Gram-negative bacteria (*Escherichia coli* ATCC 25922, *Proteus hauseri* ATCC 15442, and *Campylobacter jejuni* ATCC 33560) without adverse effects on eukaryotic cells. Both the triterpenoids showed the best antibacterial potential against the Gram-positive strains. They showed synergistic activity against all the tested microorganisms, and a bactericidal effect with the combination OA with UA against both *Staphylococcus* strains. In addition, the synergistic action of DHM, UA, and OA was reported for the first time in this study. Our results also showed that combination with triterpenoids enhanced the antimicrobial potential of DHM.

## 1. Introduction

Collectively, humans are gradually reverting to harnessing the benefits of nature, especially plants. To maintain and improve our health, we prefer natural products, including therapeutics. Unfortunately, the excessive and inappropriate use of chemotherapeutic agents has harmful effects on human health. Moreover, the increase in the number of bacterial strains resistant to many commercially available antibiotics has drawn the attention of researchers to search for alternative healing compounds. Many have been tested as new candidate antibacterial drugs, for example, antibacterial peptides produced by microorganisms [1], bacteriophages [2], and phytocompounds [3].

Plants are an abundant source of biologically active compounds, which can be potential therapeutics or precursors for the development of new drugs. Some plants may even produce phytocompounds with antimicrobial and antioxidant activities [4]. Selected substances can inhibit the growth of popular pathogens, such as *Staphylococcus aureus*, *Staphylococcus epidermidis*, *Escherichia coli*, and *Pseudomonas aeruginosa*. Moreover, isolated components from various parts of plants showed antiviral, antitumor, anti-inflammatory, antiplatelet, and prostaglandin-inhibitory activities [5,6]. Furthermore, an increase in environmental pollution, especially from the use of antibiotics, has led to the search for safer medicinal products.

**Figure 1:**
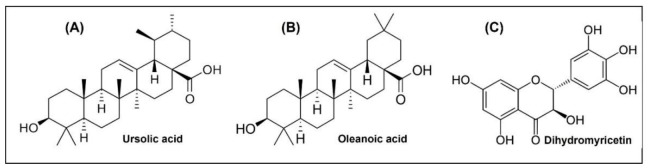
The chemical structures of (**A**) ursolic acid; (**B**) oleanoic acid; (**C**) dihydromyricetin.

Among the main components of plant extracts that have been used in natural medicine for millennia are triterpenoids, which are the most abundant group of terpenoids found in dicotyledonous plants. Although they do not play a significant role in primary metabolism, they are involved in the adaptation of plants for survival. Some triterpenoids function as specific chemical weapons against competitive plants, pathogens, or herbivores. Previous studies have described the antioxidant, antimicrobial, antiallergic, antidiabetic [7], fungicidal [8], antiparasitic [9], and anticancer [10,11] potential of triterpenes. Other studies have also reported their anti-inflammatory [12], analgesic, hepatoprotective [13,14], cardiotonic, and sedative activity [15]. Triterpenoids are synthesised in plants via squalene cyclisation, which is a C30 hydrocarbon [16]. Based on their structural skeleton, triterpenes are classified into several groups: cucurbitanes, cycloartanes, dammaranes, euphanes, fiedelanes, holostanes, hopanes, isomalabaricanes, lanostanes, lupanes, oleananes, protostanes, tirucallanes, and ursanes [17]. The representative pentacyclic triterpenoids are oleanolic acid (OA) and ursolic acid (UA). They are commonly found in nature in free acid form or as an aglycone precursor for a triterpenoid called saponin [14]. The chemical structures of these phytocompounds are shown in Figure 1A–C. These pentacyclic triterpenoids affect the expression of bacterial genes involved in biofilm formation, peptidoglycan turnover, and cell autolysis [18]. In addition, OA and UA along with their derivatives have strong antimutagenic effects [19]. Owing to their similar structures, UA and OA often occur simultaneously. In addition, the activities of these triterpenes also include their potential to enhance bacterial susceptibility to other compounds [20].

Dihydromyricetin (DHM) belongs to the flavonoid family, whose chemical structure is shown in Figure 1C. It is a major secondary metabolite of the plant *Ampelopsis grossedentata*, which is widely distributed in the mountainous areas of China and has been used in herbal medicine for centuries [21]. Previous studies have shown many valuable properties of this compound, including antioxidant [21], anti-inflammatory [22], antibacterial [23,24,25,26], neuroprotective [27], anticancer [28], and metabolic regulation of glucose and lipids [29]. It is apparent that DHM has a wide range of prospects in the food industry as an antioxidant and antibacterial agent. However, the antimicrobial mechanism of this compound has not been adequately investigated [30]. Previous reports have confirmed that hydroxylation at positions 5 and 7 of their structure can play a key role in the antibacterial activity of flavanols, and that hydroxylation of the B and C rings can enhance it [31].

However, there are many contrasting data in the literature. The reason for this may be the test methods used, the purity of the phytocompound, and/or the bacterial strain used.

In addition to the possibility of using phytochemicals in medicine, their application in the food industry is also of interest, where food-borne diseases still pose a threat. In recent years, synthetic preservatives have been commonly used in food because of their low cost and high antimicrobial activity. However, increasing consumer demand for safe, unprocessed foods and prolonged storage time is mobilizing the food industry to introduce natural antimicrobial components as synthetic preservative replacements.

The aim of this study was to investigate the antibacterial activity of phytocompounds (UA, OA, and DHM) individually and in various combinations. Finally, the cytotoxic activity of the phytocompounds was determined in vitro using mammalian cells.

## 2. Results and Discussion

### 2.1. Antimicrobial Activity of Phytocompunds

**Figure 2:**
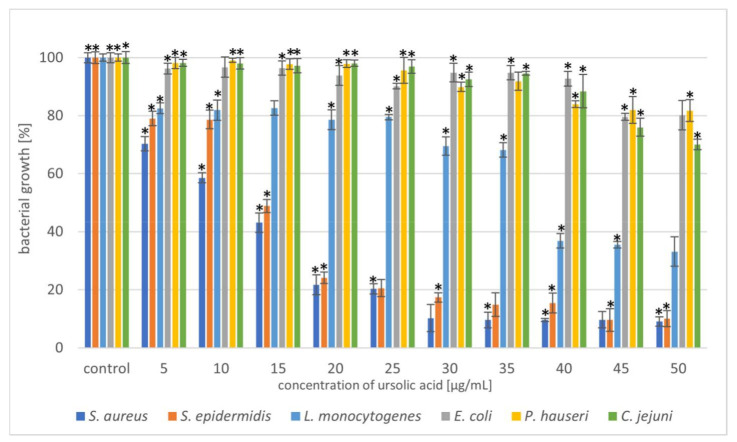
Reduction in bacterial growth after ursolic acid treatment and incubation for 24 h. The data represent mean ± SD of the three different experiments performed in triplicate. *p*-values were determined by one-way analysis of variance (ANOVA), where (*) represents statistically significant results (*p* ≤ 0.05).

**Figure 3:**
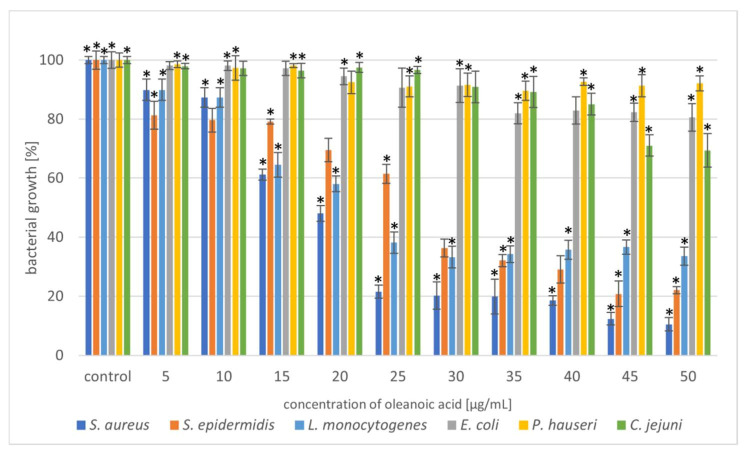
Reduction in bacterial growth after oleanoic acid treatment and incubation for 24 h. The data represent mean ± SD of three different experiments performed in triplicate. *p*-values were determined by one-way analysis of variance (ANOVA), where (*) represents statistically significant results (*p* ≤ 0.05).

**Figure 4:**
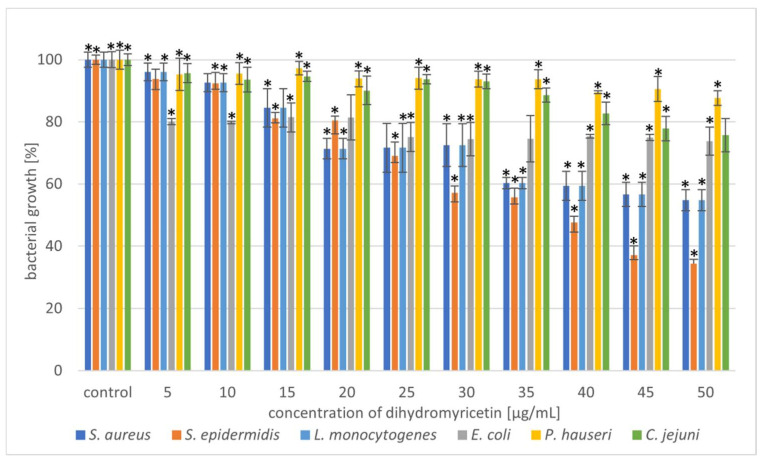
Reduction in bacterial growth after dihydromyricetin treatment and incubation for 24 h. The data represent mean ± SD of three different experiments performed in triplicate. *p*-values were determined by ANOVA where (*) represents statistically significant results (*p* ≤ 0.05).

Selected phytocompounds were tested for antimicrobial activity against six bacterial strains (*S. aureus*, *S. epidermidis*, *L. monocytogenes*, *E. coli*, *P. hauseri*, and *C. jejuni*) using the microdilution method. The antibacterial potential of UA, OA, and DHM is shown in Figure 2, Figure 3 and Figure 4 and Table S1.

Triterpenoids showed great activity toward Gram-positive strains. The addition of UA (20 µg/mL) reduced the growth of the Gram-positive strains *S. aureus* and *S. epidermidis* by 80%. In the case of the Gram-negative strain, *L. monocytogenes*, a higher concentration (40 µg/mL) of the compound resulted in 60% growth inhibition (Figure 2). Moreover, we also noted that OA was more effective against Gram-positive bacteria (Figure 3). These similar effects of the tested triterpenoids may be due to their related structures. However, higher concentrations of the compound had to be added, compared to UA. Previous studies have demonstrated high antibacterial activity of UA [15,32,33] and its derivatives [34] against *S. aureus*. Methicillin-resistant *S. aureus* (MRSA) infections are a serious problem in hospitalised patients. Olean-27-carboxylic acid-type triterpenes possess antibacterial activity against various MRSA strains as well as quinolone-resistant *S. aureus* [35]. In addition, the antimicrobial potential of UA and OA against *L. monocytogenes* have also been confirmed [36,37]. However, Panizzi et al. [38] described the lack of OA (from *Geum rivale*) activity against *S. aureus*, *E. coli*, and *P. aeruginosa*. Similarly, Calis et al. [39] did not observe antibacterial activity of OA (from *Cyclamen mirabile*) against Gram-positive (*S. aureus* and *E. faecalis*) and Gram-negative (*P. aeruginosa* and *E. coli*) bacteria. The diversity of the antimicrobial properties of UA and OA has also been observed against *Mycobacterium tuberculosis*, which is the most common cause of deaths worldwide [40,41]. In addition, bacteria, such as *E. faecalis* [42], *Streptococcus pneumoniae* [33], *E. faecium*, *Bacillus subtilis*, and *B. cereus* [32,42,43] are sensitive to OA treatment. Cunha et al. [43] investigated the antimicrobial effect of OA against *Streptococcus strains* (*S. mutans*, *S. sanguis*, *S. mitis*, and *S. salivarius*) and determined that both the hydroxy and carboxy groups in triterpenes were responsible for OA antimicrobial activity. It has been proven that the tested pentacyclic triterpenoids affect peptidoglycan structure, gene expression, and biofilm formation [44,45] in bacteria. Another mechanism of action can be associated with the induction of stress response. Grudniak et al. [46] showed that *E. coli* treated with OA altered the synthesis of DnaK, thus inducing a heat-shock response in this species. When Zhou et al. [47] used TEM to analyse the morphological changes in MRSA cells after treating them with UA and oxacillin, they observed cell membrane disintegration, cell lysis, and cytoplasmic content release.

In the present study, the triterpenoids showed less activity toward Gram-negative bacteria. The addition of UA (50 µg/mL) limited the growth of *E. coli*, *P. hauseri*, and *C. jejuni* by 20–30%, whereas that of OA (50 µg/mL) inhibited the growth of the Gram-negative strains by 10–30%. Some studies have described poor UA activity against *E. coli* [48]. Mallavadhani et al. [49] showed moderate activity of UA and its lipophilic 3-O fatty acid ester chains (C12-C18) against Gram-negative strains (*E. coli*, *S. typhi*, and *P. syringae*). Interestingly, it has been reported that UA (50 mg/mL) could be a therapeutic agent for treating *Helicobacter pylori* infections [50].

Based on the results shown in Figure 2 and Figure 3, the triterpenoids were more active against Gram-positive bacteria than Gram-negative bacteria. This may be due to the structural differences in the cell walls of these bacterial classes. Cells of Gram-negative bacteria are surrounded by an additional outer membrane, which provides them with a hydrophilic surface that functions as a permeability barrier against many substances, including natural compounds [51,52]. Moreover, the intrinsic resistance in Gram-negative bacteria is supported by efflux pumps which pump many compounds, such as toxins and antibiotics from the periplasm to the outside of the cell [53,54].

The next part of our experiment investigated the antibacterial potential of DHM against the tested bacterial strains. The results showed that with an increase in DHM concentration, the inhibition level increased. The strain most sensitive to the action of DHM was *S. epidermidis*. At a concentration of 45–50 µg/mL, growth inhibition was 65%. Other Gram-positive bacteria showed moderate sensitivity to the phytocompounds, but only at higher concentrations (35–50 µg/mL). Wu et al. [23] has previously described the antibacterial potential of DHM against *S. aureus*. Moreover, we observed that the Gram-negative strains were weakly susceptible to DHM at each concentration range. Addition of DHM at a concentration of 50 µg/mL caused a 26%, 13%, and 24% growth reduction of *E. coli*, *P. hauseri*, and *C. jejuni*, respectively. Xiao et al. [55] demonstrated that DHM has great antibacterial activity against tested food-borne bacteria (*S. aureus, B. subtilis*, *E. coli*, *S. paratyphi*, and *P. aeruginosa*). Moreover, SEM analysis (*E. coli* and *S. aureus*) suggested that DHM induces aggregation, shrivelling, and adhesion of bacteria. Cui et al. [56] performed SEM analysis of *E. coli* treated with DHM and reported that the cell had a wrinkled surface and was lysed at both ends. Apart from disturbing the integrity of the cellular membrane, DHM can also inhibit the respiratory metabolism of bacteria [55].

### 2.2. Combined Antimicrobial Activity of Selected Phytocompounds

Given the issue of multidrug-resistant strains, new ways to eliminate these pathogens must be developed, such as combinatorial therapy using phytocompounds. Therefore, studying the synergistic effects of the compounds has become a key step in phytochemical studies [57,58].

**Figure 5:**
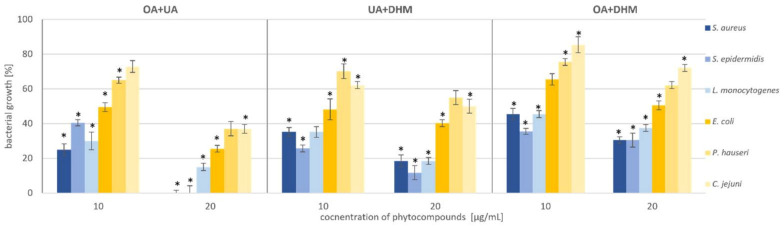
Synergistic effect of phytocompounds (OA+UA; UA+DHM; OA+DHM) against Gram-positive (*S. aureus*, *S. epidermidis*, and *L. monocytogenes*) and Gram-negative (*E. coli*, *P. hauseri*, and *C. jejuni*) bacteria. The data represent mean ± SD of the three different experiments performed in triplicate. *p*-values were determined by ANOVA, where (*) represents statistically significant results (*p* ≤ 0.05).

Therefore, we tested the antimicrobial synergy of the selected phytocompounds against the test strains. The results are presented in Figure 5. The most representative synergistic effect was observed by the treatment of 20 µg/mL OA in combination with 20 µg/mL UA against both the *Staphylococcus* strains, with bactericidal effect (Figure 5, Table 1, Table S2). Based on the previous experiment, we found that DHM had the weakest effect on the tested bacteria (Figure 4, Table 1). Our results showed that combination with triterpenoids (UA or OA) enhanced the antimicrobial potential of DHM. Coadministration of UA and DHM (20 µg/mL each) considerably reduced the growth of Gram-negative strains. The addition of both UA and DHM reduced bacterial growth by 60% for *E. coli* and approximately 45% for *P. hauseri* and *C. jejuni* (Figure 2 and Figure 4, Table 1). However, when UA and DHM were tested individually, bacterial growth was inhibited by only 7% and 6%, respectively (Figure 5, Table 1). In the case of Gram-negative strains, treatment with phytocompounds (10 and 20 µg/mL) alone did not inhibit bacterial growth. Thus, only a combination of these compounds yielded satisfactory results.

We concluded that the most promising results of synergistic effects were obtained in a combination of UA and OA. The data also indicated the possibility of reducing the dosage of the compounds.

### 2.3. Assessment of the Cytotoxic Activity of Selected Phytocompounds

**Figure 6:**
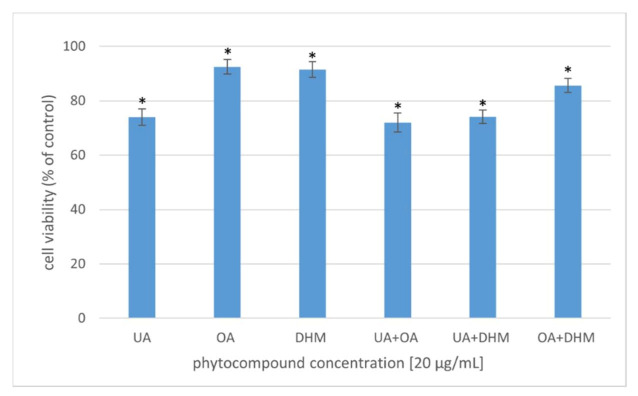
Viability of human fibroblasts after 24 h incubation with the phytocompounds [20 µg/mL]. The results were expressed as a percentage of viability of the untreated cells. The data represent mean ± SD of the three different experiments performed in triplicate. *p*-values were determined by ANOVA, where (*) represents statistically significant results (*p* ≤ 0.05).

A safe antibacterial agent should be nontoxic to eukaryotic cells and show robust activity against microorganisms. The cytotoxic activity of the phytocompounds was studied in several variants at 20 µg/mL concentration (Figure 6). The effect of UA, OA, and DHM (individual or simultaneous addition to the bacterial culture) on the viability of human fibroblasts was assessed via the MTT assay. The percentage of viable cells was computed relative to that of the control (cells incubated without phytocompounds), the viability of which was considered 100%. After 24 h of incubation of the cells with the phytocompounds alone, cell viability slightly decreased to 90% after OA and DHM treatment and to 74% after UA treatment. Fibroblast viability reduced to 72%, 74%, and 85% in the cell cultures supplemented with two of the compounds (synergistic effect): UA+OA, UA+DHM, and OA+DHM, respectively. Wójciak-Kosior et al. [59] analysed the cytotoxic activity of UA and OA against human skin fibroblasts and reported higher cytotoxic activity of UA compared to that of OA. A similar relationship was observed in the present study. Zhang et al. [60], however, reported no cytotoxic effect of UA against HCT-8 and Bell-7402 cell lines. DHM has been shown to exhibit selective cytotoxicity against non-small-cell lung cancer cells (A549 and H1975) but not against normal cells (WI-38) [61] (Kao et al., 2017). DHM has also been shown to be noncytotoxic to normal hepatocytes [62,63], but it inhibited hepatocellular carcinoma (HCC) cell proliferation and triggered apoptosis in a p53-dependent manner [63].

## 3. Materials and Methods

### 3.1. Reagents

Phytocompounds (HPLC purity ≥ 98%), including UA, OA, and DHM, were purchased from Sigma-Aldrich (St. Louis, MO, USA). 3-(4,5-dimethylthiazol-2-yl)-2-5-diphenyltetrazolium bromide (MTT) was purchased from Sigma-Aldrich (Darmstadt, Germany). The human fibroblast BJ (CRL-2522) cell line was purchased from the American Type Culture Collection (ATCC^®^, Manassas, VA, USA). Dulbecco’s Modified Eagle Medium (DMEM) and Fetal Bovine Serum (FBS) were obtained from BioWest (Nuaillé, France). DMSO was purchased from BioShop (Burlington, ON, Canada).

### 3.2. Determination of Antimicrobial Activity

The antimicrobial activity of UA, OA, and DHM was evaluated using the microdilution method for aerobic and anaerobic bacterial strains according to the CLSI documents M07 (11th Edition) [64] and M11 (9th Edition) [65], respectively. The antimicrobial activity of the phytocompounds was determined against aerobic bacteria (*S. aureus* ATCC 6538, *S. epidermidis* ATCC 12228, *E. coli* ATCC 25922, and *P. hauseri* ATCC 15442) and anaerobic bacteria (*L. monocytogenes* ATCC 19115 and *C. jejuni* ATCC 33560). The growth of the aerobic and anaerobic bacterial strains that were either treated with the phytocompounds or left untreated was evaluated in 96-well microtiter plates in Mueller–Hinton broth and Brucella broth supplemented with hemin, vitamin K_1_, and laked horse blood, respectively. The phytocompounds were supplemented in a range of 5–50 µg/mL, and their antimicrobial potential was determined. They were diluted in the appropriate growth medium before administration. An inoculum of bacteria grown in the Mueller–Hinton or Brucella broths was added to each well to achieve a final density of 5 × 10^5^ CFU/mL and 1 × 10^6^ CFU/mL for the aerobic and anaerobic strains, respectively. The microtiter plates were then incubated for 24 h at 37 °C (aerobic strains) and for 48 h at 37 °C (anaerobic strains). The plates inoculated with anaerobic strains were incubated in jars where anaerobic conditions were achieved using GasPak envelopes and monitored with a disposable BBL dry anaerobic indicator strip (Becton Dickinson). After incubation, the optical density was measured spectrophotometrically at 620 nm. Experiments for each type of phytocompound were performed in triplicate, leading to the analysis of six independent experiments. The antimicrobial activity of the tested compounds was calculated as the percentage of bacterial growth inhibition (SD) compared to that of the biotic control (bacteria incubated in the medium).

### 3.3. Determination of Cytotoxic Activity of Phytocompounds

The cytotoxic potential of phytocompounds against human cells was examined using the fibroblast BJ ATCC CRL-2522 cell line. Cells were suspended in Dulbecco’s modified Eagle medium (DMEM) containing foetal bovine serum (10%) and antibiotics (penicillin 100 IU/mL and streptomycin 100 μg/mL) at a final density of 1 × 10^5^ cells/well. The final volume of the culture was 100 μL. The fibroblasts were incubated in 96-well microplates in a humidified atmosphere at 37 °C and 5% CO^2^ for 24 h. After incubation, exhausted DMEM was replaced with fresh medium and phytocompounds were supplemented at 10–20 μg/mL. Adequate control cultures that were untreated with the selected phytocompounds were also prepared and the plates were incubated under the same conditions. After 24 h of incubation, the media were removed from the cells and the wells were supplemented with 500 μg/mL MTT. Following a 2 h incubation under the same conditions, the solution was removed from the wells, which were then refilled with 100 μL of sterile DMSO to dissolve the formazan crystals. The viability of fibroblasts was calculated based on the spectrophotometric quantification of the cultures at λ = 550 nm using a SpectraMax i3x multimode microplate reader (Molecular Devices Ltd., Wokingham, Berkshire, UK). The results are presented as the mean values of the percentages of fibroblast viability in control cultures untreated with phytocompounds with the SD. The experiment, with *n* = 4, was performed in triplicate.

In addition, the combined effects of the tested compounds on the bacteria were investigated. This allowed for the selection of the best combination of two compounds, which could allow for reduction in dosage.

## 4. Conclusions

Nature is a rich source of plant resources that possess bioactive phytocompounds. As such, medicinal phytocompounds are one of the best sources for obtaining new therapeutics that would be clinically effective, biodegradable, and safe for human use. The present work enabled us to document the antibacterial activity of UA, OA, and DHM individually and in combination with each other to test for synergy. Triterpenoids (UA and OA) proved to be successful antibacterial agents, particularly in Gram-positive strains, and considering the cytotoxicity results, all the tested phytocompounds would be safe for use in the pharmaceutical industry.

## Figures and Tables

**Table 1:** Antibacterial activity of the tested phytocompounds: ursolic acid, oleanoic acid, dihydromyricetin against *Staphylococcus aureus* ATCC 6538 (*S. a*.), *Staphylococcus epidermidis* ATCC 12228 (*S. e*.), *Listeria monocytogenes* ATCC 19115 (*L. m*.), *Escherichia coli* ATCC 25922 (*E. c.*), *Proteus hauseri* ATCC 15442 (*P. h*.) and *Campylobacter jejuni* ATCC 33560 (*C. j.*) after phytocompound treatment and incubation for 24 h.

Phytocompound Concentration [µg/mL]	Bacterial Growth [%]						Phytocompound
	*S. a.*	*S. e.*	*L. m.*	*E. c.*	*P. h.*	*C. j.*	
10	87.2	79.6	87.2	98.1	97.3	97.1	Oleanoic Acid
20	48	69.5	58	94.5	92.4	97.5	
10	58.6	78.6	81.9	96.7	99	98	Ursolic Acid
20	21.7	24.1	78.6	93.9	97.9	98	
10	92.6	92.4	92.6	79.7	95.5	93.6	Dihydromyricetin
20	71.3	80.4	71.3	81.4	93.8	90	

## Data Availability

The data presented in this study are available on request from the corresponding author.

## Supplementary Materials

The following supporting information can be downloaded. Table S1: Antibacterial activity of the tested phytocompounds: ursolic acid, oleanoic acid, dihydromyricetin against *Staphylococcus aureus* ATCC 6538 (*S. a.*), *Staphylococcus epidermidis* ATCC 12228 (*S. e.*), *Listeria monocytogenes* ATCC 19115 (*L. m*.), *Escherichia coli* ATCC 25922 (*E. c.*), *Proteus hauseri* ATCC 15442 (*P. h.*) and *Campylobacter jejuni* ATCC 33560 (*C. j*.) after phytocompound treatment and incubation for 24 h. Table S2: Synergistic antibacterial activity of the tested phytocompounds: ursolic acid, oleanoic acid, dihydromyricetin against *Staphylococcus aureus* ATCC 6538 (*S. a.*), *Staphylococcus epidermidis* ATCC 12228 (*S. e.*), *Listeria monocytogenes* ATCC 19115 (*L. m.*), *Escherichia coli* ATCC 25922 (*E. c.*), *Proteus hauseri* ATCC 15442 (*P. h.*) and *Campylobacter jejuni* ATCC 33560 (*C. j.*) after phytocompound treatment and incubation for 24 h.
