# Supplementary material for: The Synergistic Effect of Triterpenoids and Flavonoids—New Approaches for Treating Bacterial Infections?
Source: Molecules. 2022 Jan 27;27(3):847. doi: 10.3390/molecules27030847 (PMC8838219; doi:10.3390/molecules27030847)
Supplement: Supplementary file 1 [file molecules-27-00847-s001.zip › molecules-1557885-supplementary.pdf]

Supplementary Materials

The Synergistic Effect of Triterpenoids and Flavonoids—New Approaches for Treating Bacterial Infections?

Natalia Wrońska *, Michał Szlaur, Katarzyna Zawadzka and Katarzyna Lisowska

Department of Industrial Microbiology and Biotechnology, Faculty of Biology and Environmental Protection, University of Lodz, 12/16 Banacha Street, 90-236 Lodz, Poland; n37@o2.pl (M.S.); katarzyna.zawadzka@biol.uni.lodz.pl (K.Z.); katarzyna.lisowska@biol.uni.lodz.pl (K.L.)

* Correspondence: natalia.wronska@biol.uni.lodz.pl; Tel.: +48-426354149

Table S1. Antibacterial activity of the tested phytocompounds: ursolic acid, oleanoic acid, dihydromyricetin against *Staphylococcus aureus* ATCC 6538 (*S. a.*), *Staphylococcus epidermidis* ATCC 12228 (*S. e.*), *Listeria monocytogenes* ATCC 19115 (*L. m.*), *Escherichia coli* ATCC 25922 (*E. c.*), *Proteus hauseri* ATCC 15442 (*P. h.*) and *Campylobacter jejuni* ATCC 33560 (*C. j.*) after phytocompound treatment and incubation for 24 h.

	URSOLIC ACID						OLEANOIC ACID						DIHYDROMYRICETIN					
	Growth Inhibition [%]																	
**Phytocompound Concentration [μg/mL]**	*S. a.*	*S. e.*	*L. m.*	*E. c.*	*P. h.*	*C. j.*	*S. a.*	*S. e.*	*L. m.*	*E. c.*	*P. h.*	*C. j.*	*S. a.*	*S. e.*	*L. m.*	*E. c.*	*P. h.*	*C. j.*
5	29.7	21	17.5	3.8	1.8	1,8	10.1	18.7	10	1.9	1.5	2	3.9	6.2	3.9	19.8	4.7	4.4
10	41.5	21.4	18.1	3.3	1	1,9	12.8	20.4	12.8	1.8	2.6	2.8	7.4	7.5	7.4	20	4.5	6.2
15	57	51.1	17.4	3.7	2.3	2,5	38.8	20.9	35.5	2.8	2.1	3.4	15.4	18.9	15.4	18.7	3.2	5.9
20	78.3	75.8	21.4	6.1	2.3	2	52	30.5	42	5.5	7.5	2.7	28.6	19.5	28.6	18.8	6.1	9.7
25	79.7	79.5	20.5	9.85	4.4	3,1	78.4	38.5	61.8	9.4	8.8	3.4	28.5	30.8	28.3	24.9	6	7
30	89.7	82.6	30.5	5.2	10.1	7,5	79.7	63.6	66.7	8.7	8.5	9.1	27.9	42.8	27.6	25.6	6.3	7.2
35	90.4	85.2	31.9	5.3	9.7	5,4	80.1	67.9	66.1	18.1	10	10.8	39.7	44.2	39.7	25.5	6.3	11.4
40	90.4	84.6	63.1	7.3	16.4	11,6	81.4	70.9	64.5	17.2	7.8	14.9	40.5	52.4	40.6	24.6	10.5	17.2
45	90.3	90.4	64.6	20.4	18.4	24,1	87.6	79	63.5	17.7	8.6	28.9	43.4	62.9	43.4	25	9.6	22.1
50	91	90	66.9	20	18.3	30	89.5	78.1	66.4	19.4	8.1	30.6	45.2	65.7	45.2	26.2	12.4	24.3

Table S2. Synergistic antibacterial activity of the tested phytocompounds: ursolic acid, oleanoic acid, dihydromyricetin against *Staphylococcus aureus* ATCC 6538 (*S. a.*), *Staphylococcus epidermidis* ATCC 12228 (*S. e.*), *Listeria monocytogenes* ATCC 19115 (*L. m.*), *Escherichia coli* ATCC 25922 (*E. c.*), *Proteus hauseri* ATCC 15442 (*P. h.*) and *Campylobacter jejuni* ATCC 33560 (*C. j.*) after phytocompound treatment and incubation for 24 h.

**Phytocompound Concentration [μg/mL]**	*Growth Inhibition [%]*						*Phytocompounds Combination*
	S. a.	*S. e.*	*L. m.*	*E. c.*	*P. h.*	*C. j.*	
10	75	59.5	70	50.5	35	27.5	**OLEANOIC ACID + URSOLIC ACID**
20	100	100	85	74.5	62.5	63	
10	64.8	74.3	64.8	51.8	28.5	37.9	**URSOLIC ACID + DIHYDROMYRICETIN**
20	81.5	88.2	81.5	59.7	45	49.9	
10	54.5	64.5	54.5	34.5	24.6	14.5	**OLEANOIC ACID + DIHYDROMYRICETIN**
20	69.5	69.5	62.5	49.5	37.8	27.8	
